# Real-life efficacy of generic sofosbuvir/ledipasvir for treatment of Iranian patients with chronic hepatitis C: A cohort study

**DOI:** 10.22088/cjim.11.1.41

**Published:** 2020

**Authors:** Heidar Sharafi, Seyed Hoda Alavian, Bita Behnava, Mohammad Saeid Rezaee-Zavareh, Mehri Nikbin, Seyed Moayed Alavian

**Affiliations:** 1 Baqiyatallah Research Center for Gastroenterology and Liver Diseases, Baqiyatallah University of Medical Sciences, Tehran, Iran; 2 Middle East Liver Diseases (MELD) Center, Tehran, Iran

**Keywords:** Direct-acting antiviral agent, Hepatitis C, Ledipasvir, Sofosbuvir, Treatment

## Abstract

**Background:**

Treatment of hepatitis C virus (HCV) infection with recently introduced direct-acting antiviral agents (DAA) is effective and safe, however there is little known regarding safety and efficacy of generic DAAs in the real-life clinical setting. This study aimed to evaluate the efficacy and safety of generic sofosbuvir/ledipasvir (SOF/LDV) in a real-life clinical experience.

**Methods:**

In this prospective cohort study, patients with chronic HCV infection who referred to Middle East Liver Diseases (MELD) Center were included. Based on the patients’ condition, they were treated with SOF/LDV fixed-dose combination with or without ribavirin (RBV) for 12 or 24 weeks.

**Results:**

A total of 30 (M/F: 19/11) patients with chronic HCV genotype 1 infection with a mean age of 49.8 years were treated with generic SOF/LDV with (9 patients) or without (11 patients) RBV for 12 (27 patients) or 24 (3 patients) weeks. Ten (33.3%) had cirrhosis and 13 (43.3%) with a previous history of treatment with interferon (IFN)-based regimens. Among the 30 patients, 26 (86.7%, 95% CI=70.3%-94.7%) achieved a rapid virologic response, 30 (100%, 95% CI=88.7%-100%) achieved the end of treatment response and 30 (100%, 95% CI=88.7%-100%) achieved a sustained virologic response. No severe treatment adverse event was observed however, 6 (20%) patients experienced mild to moderate adverse events.

**Conclusion:**

The treatment of HCV genotype 1 infection with generic SOF/LDV found to be safe and effective even in patients with cirrhosis and previous history of treatment with IFN-based treatments.

Hepatitis C virus (HCV) infection, causing around 700,000 deaths annually is recognized as a global health concern (1). Treatment-induced clearance of HCV infection can reduce the risk of developing advanced liver diseases (2). For more than a decade, the standard of care for the management of HCV was using a combination therapy with Pegylated-interferon (PegIFN) and ribavirin (RBV) however, this therapeutic regimen was not an optimal regimen for treatment of HCV infection with 40-70% treatment success rate and many side-effects (3-7). The standard of care for treatment of hepatitis C has changed gradually since 2011 with the introduction of first-generation of direct-acting antiviral agents (DAAs) and optimized with the introduction of NS5B polymerase inhibitors in 2013 and finally the introduction of first-ever all-oral IFN-free DAA-based treatment of HCV infection in 2014 (8-10).

In clinical trials using brand-name drugs, these DAA-based regimens resulted in sustained virologic response (SVR) in more than 95% of patients and rare observation of severe adverse-events specially those causing treatment discontinuation (11). Fixed-dose combination of sofosbuvir/ledipasvir (SOF/LDV) was the first all-oral IFN-free daily single tablet for the treatment of HCV genotype 1 (HCV-1) infection (11). It is worth to note that HCV-1 is the most common genotype in Iran and most parts of the world (12, 13). Fortunately, the Iranian pharmaceutical industry has manufactured the generic DAAs such as SOF/LDV with affordable prices. The real-life experience with these generic DAAs is important to show if the generic DAAs have the same effectiveness as the brand-name DAAs.

The aim of the current study was to evaluate the real-life clinical experience with generic SOF/LDV fixed-dose combination in Iranian patients with HCV-1 infection in terms of efficacy and safety.

## Methods

**Study Design and Population: **This prospective cohort study was conducted on the patients consecutively referred to Middle East Liver Diseases (MELD) Center (Tehran, Iran) for the clinical management of hepatitis C from October 2016 to June 2017. The inclusion criteria were: a. Age > 18 years old, b. Chronic HCV infection (being positive for HCV Ab and HCV RNA more than 6 months), c. HCV-1 infection. The exclusion criteria were: a. co-infections with HBV and/or HIV, b. hepatocellular carcinoma (HCC), c. HCV genotypes other than 1, d. chronic kidney disease with eGFR < 30 mL/min, e. thalassemia, f. pregnant women, g. decompensated liver disease with MELD > 20.

The Ethics Committee of the Baqiyatallah Research Center for Gastroenterology and Liver Diseases approved the study design. All study participants provided informed consent. The authors assert that all procedures contributing to this work comply with the ethical standards of the relevant national and institutional committees on human experimentation and with the Helsinki Declaration of 1975, as revised in 2008. The study has been registered in Iranian Registry of Clinical Trials (IRCT) under registration number IRCT2016050717413N15.

**Treatment Protocol and Virologic Response Definition: **The included patients were treated with a daily fixed-dose combination of SOF 400 mg/LDV 90 mg (Hepasbuvir Plus; Danesh Pharmaceutical Develpment CO., Iran). The treatment protocol was based on the national recommendations for the clinical management of hepatitis C in Iran, 2016 (8). Briefly, patients without cirrhosis were treated with 12-week regimen of SOF/LDV, patients with compensated cirrhosis (Child-Pugh A) were treated with 12-week regimen of SOF/LDV plus daily weight adjusted RBV (1000 mg for < 75 kg and 1200 mg for > 75 kg) and patients with decompensated cirrhosis (Child-Pugh B and C) were treated with 24-week regimen of SOF/LDV plus daily weight adjusted RBV (1000 mg for <75 kg and 1200 mg for >75 kg). In cases with RBV-intolerance or -contraindication, a 24-week regimen of SOF/LDV without RBV was considered for treatment of HCV infection.

The sustained virologic response was defined as undetectable HCV RNA, 12 weeks after treatment completion. Rapid virologic response (RVR) was defined as undetectable HCV RNA at week 4 of treatment and end of treatment response (ETR) as undetectable HCV RNA at the end of treatment. Recurrence of HCV RNA, 12 weeks after completion of treatment were considered as relapse and treatment failure.

**Laboratory and Liver Fibrosis Assessments and Physical Examinations: **Complete blood count, liver function tests, and HCV RNA level were assessed for the patients at baseline, week 4 of treatment, end of treatment and 12 weeks after termination of treatment. Plasma HCV RNA level was quantified by COBAS TaqMan HCV test, version 2.0 (Roche Diagnostics) with a lower limit of detection of 10 IU/mL. Cirrhosis was evaluated using transient elastography (FibroScan, Echosens), liver biopsy or based on clinical, laboratory and imaging evidence of cirrhosis.

Measurement of vital signs and symptom-directed physical examinations were done in each visit at the beginning of the study, 4 weeks after the treatment, at the end of treatment, and 12 weeks after the end of treatment. All adverse events including abdominal pain, alopecia, anemia, arthralgia, back pain, bleeding, bradycardia, chest pain, chills and fever, constipation, cough, depression, dermatitis, diarrhea, dizziness, dry skin, dyspnea, fatigue, flue-like syndrome, headache, hearing disorders, insomnia, irritability, jaundice, lethargy, malaise, myalgia, nasal congestion, nausea, palpitation, pruritus, rash, reflux, skin lesion, tachycardia, thrombocytopenia, vision disorders, vitiligo, vomiting, weight gain, weight loss and weakness were recorded.

**Statistical Analysis: **Categorical variables were expressed by frequency and percentage. Continuous variables with normal distribution were expressed by mean±standard deviation (SD) and continuous variables deviated from normal distribution by median (interquartile range). All statistical analyses were performed using SPSS version 22 and the statistical graph was generated using GraphPad Prism version 6.

## Results

**Patients’ Baseline Characteristics: **In this study, 30 patients with HCV infection were included and treated with generic SOF/LDV fixed-dose combination with or without RBV. Most of the patients were males (63.3%), non-cirrhotic (66.7%) and had no previous history of HCV antiviral therapy (56.7%). The pretreatment characteristics of the study population are represented in table 1.

**Table 1:** Baseline characteristics of the study population

		**All patients (n=30)**
Sex, n (%)	Male	19 (63.3)
	Female	11 (36.7)
Age (years),	mean±SD	49.8±13.1
	range (min-max)	30-67
BMI (Kg/m^2^),	mean±SD	24.5±5.4
	range (min-max)	16.8-42.7
Serum ALT (IU/L),	median (IQR)	51.0 (36.0)
	range (min-max)	19-148
Serum AST (IU/L),	median (IQR)	43.0 (25.0)
	range (min-max)	20-102
Cirrhosis condition, n (%)	Non-cirrhotic	20 (66.7)
	Compensated cirrhosis	9 (30.0)
	Decompensated cirrhosis	1 (3.3)
HCV RNA (Log IU/mL),	median (IQR)	6.5 (7.1)
	range (min-max)	3.4-7.8
HCV genotype, n (%)	HCV-1a	10 (33.3)
	HCV-1b	3 (10.0)
	Unsubtyped HCV-1	17 (56.7)
Previous history of treatment, n (%)	Treatment-naive	17 (56.7)
	Relapser	8 (26.7)
	Non-responder	5 (16.7)

N: number

SD: standard deviation

Min:minimum

Max:maximum

IQR: interquartile range

BMI: body mass index

ALT: alanine transaminase

AST: aspartate transaminase

**Figure 1:**
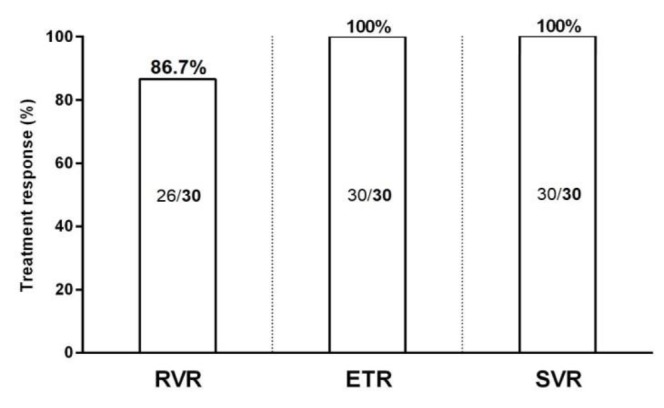
Responses to treatment with generic sofosbuvir/ledipasvir±ribavirin

RVR, rapid virologic response; ETR, end of treatment response; SVR, sustained virologic response

**Efficacy of Generic Sofosbuvir/Ledipasvir±Ribavirin: **In this study, 19 (63.3%) patients treated with SOF/LDV for 12 weeks, 8 (26.7%) with SOF/LDV+RBV for 12 weeks, 2 (6.7%) with SOF/LDV for 24 weeks and 1 (3.3%) with SOF/LDV+RBV for 24 weeks. All (100%) patients were tested for HCV RNA at week 4 of treatment (for assessment of RVR), end of treatment course (for assessment of ETR) and 12 weeks after cessation of treatment (for assessment of SVR). Among the 30 treated patients with SOF/LDV±RBV, 26 (86.7%, 95%CI=70.3%-94.7%) achieved RVR and all (100%, 95%CI=88.7%-100%) achieved both ETR and SVR. Among the 4 patients who did not achieve RVR, all were non-cirrhotic, 3 had previous history of treatment with PegIFN+RBV, 3 were treated with SOF/LDV for 12 weeks and 1 with SOF/LDV+RBV for 12 weeks and all had baseline HCV RNA level > 10^6 ^IU/mL. The response rates (RVR, ETR, and SVR) to generic SOF/LDV±RBV are presented in figure 1.

**Safety of Generic Sofosbuvir/Ledipasvir±Ribavirin: **Following treatment with generic SOF/LDV±RBV, none of the patients experienced severe treatment adverse events resulting in treatment discontinuation. Among the 9 patients who received SOF/LDV+RBV, 5 (55.6%) experienced anemia resulted in RBV-dose modification in one patient. Among the 30 patients treated with SOF/LDV±RBV, 6 (20%) experienced following mild to moderate treatment adverse events: anemia, constipation, headache, fatigue, malaise, nausea and skin lesion. The side effects of treatment with SOF/LDV±RBV are summarized in table 2.

**Table 2:** Side-effects of treatment with generic sofosbuvir/ledipasvir±ribavirin

**Side-effect**	**n (%)**
Anemia	5 (16.7)
Constipation	1 (3.3)
Headache	1 (3.3)
Fatigue	1 (3.3)
Malaise	1 (3.3)
Nausea	1 (3.3)
Skin lesion	1 (3.3)
Others*	0 (0)

*Abdominal pain, alopecia, arthralgia, back pain, bleeding, bradycardia, chest pain, chills and fever, cough, depression, dermatitis, diarrhea, dizziness, dry skin, dyspnea, flu-like syndrome, hearing disorders, insomnia, irritability, jaundice, lethargy, myalgia, nasal congestion, palpitation, pruritus, rash, reflux, tachycardia, thrombocytopenia, vision disorders, vitiligo, vomiting, weight gain, weight loss, weakness

## Discussion

This study showed that treatment of HCV infection with generic SOF/LDV regimen is extremely effective (100%) and safe in a clinical real-life experience. There is a large number of studies evaluating the efficacy and safety of treatments of HCV with brand-name DAAs published in the literature. However, the data regarding the efficacy and safety of antiviral therapy of HCV with generic DAA in a real-life setting in Iran is scarce. Previously, two studies were published on the efficacy and safety of HCV antiviral treatment with DAAs in Iran (14, 15). In the study by Merat et al. (14), 100 Iranian cirrhotic patients with hepatitis C (56 with HCV-1) were treated with a fixed-dose combination of SOF/daclatasvir plus RBV for 12 weeks. In the latter study, the response rate (per-protocol) of patients with HCV-1 infection was 98.1%. In another study, 30 Iranian patients with chronic HCV-1 or -4 infections were treated with generic SOF/LDV and 29 (96.7%) achieved SVR (15). These two studies confirm the results of the current real-life experience with SOF/LDV in terms of treatment efficacy and safety in Iranian patients with HCV-1 infection including those with advanced liver disease and/or previous history of treatment with IFN-based regimens.

In the era of IFN-based therapies, many parameters modified the treatment response including age, gender, host genetics (i.e. variations in *IFNL3* and *IFNL4* genes), HCV RNA level, HCV genotype and HCV gene variations (i.e. variations in Core and ISDR regions) (16-20). Targeting of HCV functional proteins with DAAs resulted in fading out of baseline predictors of treatment response through optimization of treatment. However, few parameters remained or emerged as the predictors of response to DAA-based therapies such as HCV genotype, cirrhosis, previous history of treatment (IFN-based or DAA-based) and resistance-associated substitutions (RAS) (11, 21, 22). Using IFN-based regimens, the strongest predictor of treatment response was on-treatment viral kinetics and response (i.e. RVR and EVR) which was implemented for response-guided therapy, however, the same was not true for DAA-based therapies (23, 24). In the current study, all 4 patients without achievement of RVR had high HCV RNA levels at baseline, however, they all achieved ETR and SVR even with a 12-week treatment course.

With the availability of affordable generic DAAs in Iran, there is a great chance to eliminate hepatitis C in Iran by 2030 (25). In addition to the high efficacy and safety of DAA-based treatments, they can be used for the treatment of special groups of HCV-infected patients with IFN and RBV contraindication or intolerance such as patients with cirrhosis, thalassemia and those with liver or kidney transplantation (25, 26). This study faced limitations such as small sample size resulted from a single-center study design to minimize the bias in the real-life clinical management of patients however a multi-centric study on the experience of HCV antiviral therapy with DAA-based regimens in Iran would be helpful. Another parameter limited our study was the unavailability of assessment of HCV NS5A RASs prior to treatment.

In conclusion, the treatment of HCV infection with generic SOF/LDV regimen is effective and safe. This SOF/LDV regimen is manufactured in Iran and seems to have the same efficacy and safety with brand-name SOF/LDV while it is available much cheaper than the brand-name SOF/LDV in Iran. With the availability of affordable generics, Iran can join the 12 countries currently on track to achieve the elimination of hepatitis C by 2030, however, it needs the commitment of policy-makers and stakeholders to have such achievement.
